# An appraisal of clinical practice guidelines for constipation: a right attitude towards to guidelines

**DOI:** 10.1186/s12876-016-0466-8

**Published:** 2016-05-04

**Authors:** Hongliang Tian, Chao Ding, Jianfeng Gong, Xiaolong Ge, Lynne V. McFarland, Lili Gu, Qiyi Chen, Chunlian Ma, Weiming Zhu, Jieshou Li, Ning Li

**Affiliations:** Department of General Surgery, Jinling Hospital, Medical School of Nanjing University, No. 305 East Zhongshan Road, Nanjing, 210002 China; Department of Medicinal Chemistry, University of Washington, Seattle, WA USA

**Keywords:** Constipation, Clinical practice guideline, AGREEII

## Abstract

**Background:**

Clinical practice guidelines (CPGs) are formally developed statements that assist users to provide proper health care for a kind of disease and play a significant contribution in healthcare system. This study report the methodological quality of CPGs on constipation.

**Methods:**

The “Appraisal of Guidelines and Research and Evaluation” (AGREEII) instrument was developed to determine the quality of CPGs. A comprehensive search was developed using five databases and three guideline websites until/up to December, 2015. Four independent authors evaluated the methodological issues of the CPGs by the AGREEII instrument.

**Results:**

We identified 22 relevant guidelines on constipation from 1234 citations. The overall agreement among evaluators was 0.84 using the intra-class correlation coefficient. The mean AGREEII scores for the domains “scope and purpose” (51.77) and “rigor of development” (56.73) were moderate; afterward, three domains “stakeholder involvement” (32.23), “editorial independence” (29.59) and “applicability” (29.14) were low scores. The “clarity and presentation” (23.73) had the lowest scores.

**Conclusion:**

Although existing constipation guidelines may accurately reflect current clinical practices, many guidelines’ methodological quality is low. Therefore, more emphasis and attentions should be taken to the development of high-quality guidelines.

## Background

Constipation is a disorder defined by incomplete defecation, and/or infrequent bowel movements which associated with persistent difficult and/or painful defecation, fecal incontinence, and abdominal pain [1]. It is a common clinical functional diseases. The worldwide constipation surveys show a wide range of prevalence rates between 1 % and >20 % in western populations, although, a recent epidemiological reports found 16 % general adult populations were constipation [2]. Constipation may be found for up to 20 % of community-dwelling elderly individuals. Moreover the incidence of functional constipation in childhood estimated 3 % [3].

Because of its high disease burden, the treatment of constipation has become an important issue for clinicians and patients. During the last two decade there were more than 20 developed clinical practice guidelines (CPGs) to manage the constipation. The main role of the CPGs is to give clear recommendations to help clinicians make appropriate clinical decision for specific clinical circumstance [4, 5].

However, not all guidelines are developed with the same methodologically rigorous approaches, there is no research for evaluating the quality of CPGs on constipation so far. With the above in mind, the objectives of the present study was to systematically review guidelines using the appraisal of guidelines for research and evaluation (AGREEII) instrument related to constipation [6].

## Methods

### Literature search

An electronic literature search using multiple databases (PubMed, EMBASE, The China Journal Full-text Database, Chinese Biomedical Literature Database, Chinese Scientific Journals Full-text Database), and guideline website or databases—including the Guidelines International Network (GIN) Database, the National Guideline Clearinghouse (NGC), National Institute for Health and Care Excellence (NICE), Scottish Intercollegiate Guidelines Network (SIGN), National Comprehensive Cancer Network (NCCN) was conducted limited to Chinese and English from the inception to May 2015. MeSH terms and text words “guideline, consensus, recommendation, criteria, statement, constipation” for constipation and guidelines were used within the MEDLINE database. The same search strategy was made applicable for the other databases or websites.

### Guideline selection and data extraction

Four reviewers (THL,DC,LN,GJF) independently extracted the guidelines which met the characteristics (for example, a clear guideline definition as proposed by the institute of Medicine [4], focused exclusively on constipation disease). We constructed a standard form table to extract the data of guidelines. Four reviewers extracted data separately, disagreements were discussed or by a fifth reviewer (GXL) if no consensus was reached.

### Quality appraisal and recommendation

We evaluated the twenty-two included CPGs quality by AGREEII instrument [6]. The instrument includes a 23-item tool comprising six quality domains. The four authors read the entire AGREEII handbook and then independently rated all included guidelines using formula as follows:

$$ \frac{\mathrm{Obtained}\ \mathrm{score}\ \hbox{-}\ \mathrm{Minimum}\ \mathrm{possible}\ \mathrm{score}}{\mathrm{Maximum}\ \mathrm{possible}\ \mathrm{socre}\ \hbox{-}\ \mathrm{Minimum}\ \mathrm{possible}\ \mathrm{score}}\times 100\% $$

According to the handbook for use of the AGREEII instrument, the six domain scores were considered independently. Finally, a guideline is labelled as “strongly recommended” if most domain scores are greater than 60 %. Guideline is “recommended” when most scores are between 30 % and 60 %. A guideline is labelled as “not recommended” when most domain scores are less than 30 % [7].

### Statistical analysis

A descriptive study of item frequency was carried out and the AGREEII domain scores calculated as means. Intra-class correlation coefficients (ICCs) is a measure of the reliability of measurements or ratings within each domain [8]. Statistically significant was considered if *p* value less than 0.05.

## Results and discussion

### Literature search

**Fig. 1:**
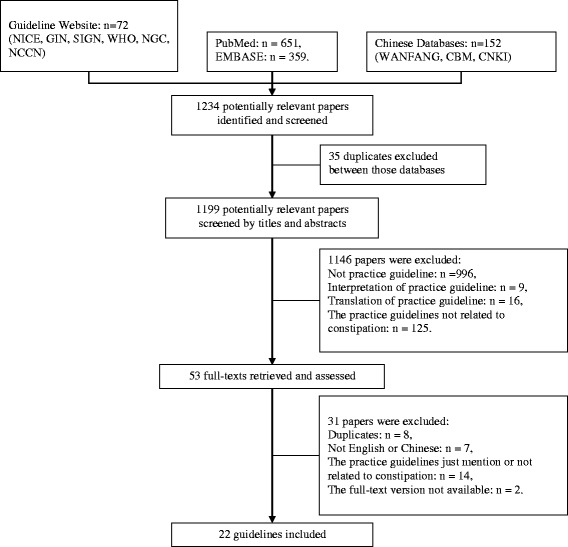
Flow of information through the different phases of the literature search

Figure 1 shows how we screened the guidelines, we preliminary search found 1234 citations, 35 were excluded because they were duplicate citations. By screening their titles and abstracts and 1,146 citations were ineligible as they didn’t meet the characteristics of constipation CPGs, 31 articles were excluded from the left 53 studies as following: eight were duplicates, seven were not in English or Chinese, 14 were guidelines not related to constipation, and 2 guidelines were the old version. Finally, a total of 22 guidelines were included [9–31].

### Guideline characteristics

The summary of CPGs baseline data were shown in Table 1. The twenty-two CPGs published between 2000 and 2014. Of the 22 selected CPGs, half of were from north America (America and Canada), six from European (UK, Ireland, Italy, Sweden) and the remaining five were from Asia (two from China, one from Korea, one from Indonesia and one multi-national),respectively. The scope of the CPRs varied: one guideline topic covered prevention, diagnosis and treatment of constipation [31]; two focused only on prevention and treatment [14, 16]; 16 covered diagnosis and treatment [9, 12, 13, 15, 17–19, 21–29]; three only focused on treatment [10, 20, 30] and one focused on prevention [11]. CPGs cited a range of number of references (range: 0–364, mean: 78) and were of varying length (mean number of pages = 25, range: 5–255). Each of the domains being evaluated using the AGREEII appraisal (Table 2). The ICCs score was moderate among raters (0.84; 95 % CI, 0.56–0.86).

**Table 1:** Characteristics of clinical practice guidelines for constipation

Title	Year	Country	Organization	Type of constipation	Topics covered	Update	No. of reference	Guideline page	Publication types	Systematic search	Evidence-based	Financial
American Gastroenterological Association Medical Position Statement: Guidelines on Constipation [9]	2000	USA	AGA	Slow-Transit Constipation, Pelvic Floor Dysfunction, Combination Syndromes	Diagnosis,treatment	Not reported	1	6	Journal	Not reported	Not reported	Not reported
Practice Guidelines for the Management of Constipation in adults [10]	2002	USA	Not reported	Constipation in adults	Treatment	Not reported	203	51	Special website (http://www.arna.com.au/)	Not reported	Not reported	Not reported
Prevention of constipation in the older adult population [11]	2005	Canada	RNAOPA	Older adult populations	Prevention	Yes	226	16	Special website (http://rnao.ca/bpg/guidelines/prevention-constipation-older-adult-population)	Not reported	Yes	Not reported
Management of chronic constipation: recommendations from a consensus panel [12]	2005	USA	None	Chronic constipation	Diagnosis,treatment	Not reported	55	8	Journal	Yes	Not reported	Yes,Pharmaceuticals Cor.
Evaluation and Treatment of Constipation in Infants and Children: Recommendations of the North American Society for Pediatric Gastroenterology, Hepatology and Nutrition [13]	2006	USA	NASPGHAN Constipation Guideline Committee	Infants and Children constipation	Diagnosis,treatment	Yes	96	13	Journal	Not reported	Yes	Not reported
Putting evidence into practice:evidence-based inventions for the prevention and management of constipation in patients with cancer [14]	2008	USA	ONS	constipation in patients with cancer	Prevention,treatment	Not reported	72	21	Journal	Not reported	Yes	Not reported
The management of constipation in palliative care: clinical practice recommendations [15]	2008	Ireland	The European Consensus Group on Constipation in Palliative Care	Constipation	Diagnosis,treatment	Not reported	43	13	Journal	Not reported	Yes	Yes,Pharmaceuticals Cor.
Management of constipation [16]	2009	UK	NICE	Older adults	Prevention,treatment	Not reported	34	10	Special website (AHRQ)	Yes	Yes	Not reported
National Consensus on The Management of Constipation in Indonesia 2010 [17]	2010	Indonesia	ISG	Constipation	Diagnosis,treatment	Not reported	18	8	Journal	Not reported	No	Not reported
Constipation in Children and Young People: Diagnosis and Management of Idiopathic Childhood Constipation in Primary and Secondary Care [18]	2010	UK	NCC-WCH	Constipation in children and young people	Diagnosis,treatment	Not reported	140	255	Special website (NICE)	Yes	Yes	Not reported
Consensus Recommendations for the Management of Constipation in Patients with Advanced, Progressive Illness [19]	2010	Canada	The Canadian Consensus Development Group	Patients with Advanced, Progressive Illness	Diagnosis,treatment	Not reported	20	13	Journal	Not reported	Yes	Yes (edu cational grant from Wyeth)
Prucalopride for the treatment of chronic constipation in women [20]	2010	UK	NICE	Chronic constipation in women	Treatment	Not reported	11	38	Special website (Cancer Care Ontario Website)	Not reported	Yes	Not reported
Common views on diagnosis and treatment of chronic constipation with Chinese medicine [21]	2011	China	CACM	Chronic constipation	Diagnosis,treatment	Yes	24	5	Journal	Not reported	No	Not reported
World Gastroenterology Organisation Global Guideline Constipation-A Global Perspective [22]	2011	Sweden	WGO	Adult patients	Diagnosis,treatment	Not reported	0	5	Journal	Not reported	Yes	None
Consensus statement AIGO/SICCR diagnosis and treatment of chronic constipation and obstructed defecation [23, 24]	2012	Italy	AIGO/SICCR	Chronic constipation and obstructed defecation	Diagnosis Treatment	Not Reported	364	30	Journal	Not reported	Yes	Associazione Italiana Gastroenterologi and Endoscopisti Digestivi Ospedalieri
Practical Treatments for Constipation in Korea [25]	2012	Korea	KSNM	Constipation	Diagnosis,,treatment	Not reported	63	9	Journal	Not reported	Not reported	Not reported
Diagnosis and Treatment Guideline of Chronic Constipation in China [26]	2013	China	CMAG/CMAS	Chronic constipation	Diagnosis,treatment	Not reported	63	8	Journal	Not reported	Not reported	Not reported
Evaluation and Treatment of Functional Constipation in Infants and Children: Evidence-Based Recommendations From ESPGHAN and NASPGHAN [27]	2013	USA	ESPGHAN and NASPGHAN	Functional Constipation in Infants and Children	Diagnosis, treatment	Yes	111	17	Journal	Yes	Yes	NASPGHAN and ESPGHAN
Primary Care Management of Chronic Constipation in Asia: The ANMA Chronic Constipation Too l [28]	2013	Asia	ANMA	Chronic Constipation	Diagnosis,,treatment	Not reported	124	12	Journal	Not reported	No	educational grant was receive d from Janssen Pharmaceuticals and Boehringer Ingelheim
American Gastroenterological Association Medical Position Statement on Constipation [29]	2013	USA	AGA	Chronic Constipation	Diagnosis,treatment	Yes	1	7	Journal	Not reported	Yes	Not reported
Lubiprostone for treating chronic idiopathic constipation [30]	2014	UK	NICE	Chronic idiopathic constipation	Treatment	Not reported	0	9	Special website (NICE)	Yes	Yes	NICE
Emerging treatments in neurogastroenterology: a multidisciplinary working group consensus statement on opioid-induced constipation [31]	2014	USA	None	Opioid-induced constipation	Prevention,diagnosis,treatment	Not reported	61	10	Journal	Not reported	Not reported	AstraZeneca

Footnotes: *NICE* National Institute for Health and Care Excellence, *ESMO* European Society for Medical Oncology, *ESDO* European Society of Digestive Oncology, *NCCN* National Comprehensive Cancer Network, *SEOM* Spanish Society of Medical Oncology, *GIN* Guidelines International Network, *NASPGHAN* North American Society for Pediatric Gastroenterology, Hepatology and Nutrition, *AIGO* Italian Association of Hospital Gastroenterologists, *SICCR* Italian Society of Colo-Rectal Surgery, *NCC*-*WCH* National Collaborating Centre for Women‘s and Children‘s Health, *KSNM* Constipation Study Group in the Korean Society of Neurogastroenterology and Motility, *NASPGHAN* the North American Society for Pediatric Gastroenterology Hepatology and Nutrition, *ESPGHAN*: the European Society for Pediatric Gastroenterology, Hepatology, and Nutrition, *ANMA* the Asian Neurogastroenterology and Motility Association, *AGA* American Gastroenterological Association, *RNAOPA* Registered Nurses Assocation of Ontario-Professional Association, *ONS* the Oncology Nursing Society, *ISG* The Indonesian Society of Gastroenterology, *CACM* China Association of Chinese Medicine, *WGO* World Gastroenterology Organization, *CMAG/CMAS* Chinese Medical Association of Gastroenterology Branch, Chinese Medical Association of Surgery Branch

**Table 2:** Guideline score according to score on each of the domains assessed by the AGREEII instrument

Title	Scope and Purpose	Stakeholder	Rigour	Clarity	Applicability	Editorial Independence	Recommendation
American Gastroenterological Association Medical Position Statement: Guidelines on Constipation [9]	31 %	11 %	7 %	44 %	15 %	0 %	Not recommended
Practice Guidelines for the Management of Constipation in adults [10]	44 %	22 %	36 %	44 %	25 %	0 %	Not recommended
Prevention of constipation in the older adult population [11]	81 %	39 %	58 %	72 %	54 %	54 %	Strongly recommended
Management of chronic constipation: recommendations from a consensus panel [12]	39 %	14 %	9 %	53 %	17 %	33 %	Not recommended
Evaluation and Treatment of Constipation in Infants and Children: Recommendations of the North American Society for Pediatric Gastroenterology, Hepatology and Nutrition [13]	56 %	31 %	46 %	58 %	31 %	0 %	Recommended
Putting evidence into practice:evidence-based inventions for the prevention and management of constipation in patients with cancer [14]	64 %	28 %	45 %	47 %	33 %	0 %	Recommended
The management of constipation in palliative care: clinical practice recommendations [15]	56 %	17 %	41 %	67 %	23 %	21 %	Not recommended
Management of constipation [16]	75 %	31 %	63 %	67 %	44 %	29 %	Recommended
National Consensus on The Management of Constipation in Indonesia 2010 [17]	56 %	8 %	3 %	69 %	10 %	0 %	Not recommended
Constipation in Children and Young People: Diagnosis and Management of Idiopathic Childhood Constipation in Primary and Secondary Care [18]	78 %	61 %	50 %	75 %	54 %	71 %	Strongly recommended
Consensus Recommendations for the Management of Constipation in Patients with Advanced, Progressive Illness [19]	50 %	19 %	40 %	64 %	21 %	50 %	Recommended
Prucalopride for the treatment of chronic constipation in women [20]	56 %	39 %	33 %	72 %	58 %	50 %	Recommended
Common views on diagnosis and treatment of chronic constipation with Chinese medicine [21]	17 %	8 %	11 %	56 %	23 %	0 %	Not recommended
World Gastroenterology Organisation Global Guideline Constipation-A Global Perspective [22]	36 %	14 %	14 %	47 %	21 %	83 %	Not recommended
Consensus statement AIGO/SICCR diagnosis and treatment of chronic constipation and obstructed defecation [23, 24]	50 %	19 %	48 %	50 %	21 %	17 %	Not recommended
Practical Treatments for Constipation in Korea [25]	44 %	11 %	16 %	39 %	21 %	33 %	Not recommended
Diagnosis and Treatment Guideline of Chronic Constipation in China [26]	25 %	11 %	15 %	47 %	19 %	0 %	Not recommended
Evaluation and Treatment of Functional Constipation in Infants and Children: Evidence-Based Recommendations From ESPGHAN and NASPGHAN [27]	81 %	25 %	66 %	83 %	23 %	38 %	Recommended
Primary Care Management of Chronic Constipation in Asia: The ANMA Chronic Constipation Tool [28]	50 %	36 %	22 %	53 %	23 %	38 %	Recommended
American Gastroenterological Association Medical Position Statement on Constipation [29]	33 %	17 %	25 %	61 %	42 %	25 %	Not recommended
Lubiprostone for treating chronic idiopathic constipation [30]	75 %	39 %	46 %	44 %	38 %	67 %	Recommended
Emerging treatments in neurogastroenterology: a multidisciplinary working group consensus statement on opioid-induced constipation [31]	42 %	22 %	15 %	36 %	25 %	42 %	Not recommended
Total ($\overline{X}$±SD)	51.77 ± 18.24	23.73 ± 13.16	32.23 ± 19.24	56.73 ± 12.91	29.14 ± 13.55	29.59 ± 25.91	-

### Appraisal of guidelines

#### Domain 1

Scope and purpose is concerned with the overall aim of the guideline, the specific health questions, and the target population (items 1–3) [32]. This domain’s mean score was 51.77 %, and nine of the guidelines (47.62 %) scored below 50 % [9, 10, 12, 21, 22, 25, 26, 29, 31].

#### Domain 2

Stakeholder involvement focuses on the extent to which the guideline was developed by the appropriate stakeholders and represents the views of its intended users (items 4–6) [32]. Of all AGREEII domains, this domain received the lowest scores (23.73 %) with only one CPG scoring over 50 %. Eighteen CPGs had been developed by a multi-disciplinary organization (81.82 %) [9, 11, 13–30].

#### Domain 3

Rigor of development criteria relates to the process used to gather and synthesize the evidence, the methods to formulate the recommendations, and to update those (items 7–14) [32]. Overall, the mean score for this domain was only 32.23 % (range, 3 % to 66 %), with 18 CPGs scoring < 50 %. Meanwhile, only five CPGs reported systematic evidence searching [12, 16, 18, 27, 30], and Just 40.90 %(9/22) guidelines provided the methods for formulating the recommendations [11–16, 26, 27, 29]. Moreover, an explicit link between the recommendations and the evidence were explicit in 20/22 of the guidelines and only five guidelines described a procedure about updating [11, 13, 21, 27, 29].

#### Domain 4

Clarity of presentation deals with the language, structure, and format of the guideline (items 15–17) [32]. The mean score for this domain was 56.73 % (range, 36 % to 83 %). Most CPGs provided a concrete and precise description of key recommendations with only eight guidelines scoring less than 50 % [9, 10, 14, 22, 25, 26, 30, 31].

#### Domain 5

Applicability pertains to the likely barriers and facilitators to implementation, strategies to improve uptake, and resource implications of applying the guideline (items 18–21) [32]. This domain’s score was 29.14 % (range, 10 % to 58 %) and only three CPGs scored > 50 % [11, 18, 20]. A total of 10 CPGs discussed barriers to implementing the guideline’s recommendations [11, 13, 14, 16, 18, 20, 21, 29–31] and 7 guideline provides advice and/or tools on how the recommendations can be put into practice [11, 16, 18, 20, 29–31]. Resource implications were not explicitly discussed, only five CPGs offered cost implications [11, 18, 20, 29, 30].

#### Domain 6

Editorial independence is concerned with the formulation of recommendations not being unduly biased with competing interests (items 22–23) [32]. The mean score for this domain was 29.59 %. Fifteen guidelines scored below 50 %. Most (63.64 %) guidelines did not provide the information whether they received funding or not [9–11, 13, 14, 16–18, 20–22, 25, 26, 29].

### Overall assessment

Guidelines were graded by the overall assessment. Only two CPGs can be strongly recommended [11, 18]. Eight can be recommended with provisions or alterations because of the most domains scoring between 30 % and 60 % [13, 14, 16, 19, 20, 27, 28, 30]. The remaining 12 CPGs were labelled as ‘not recommended’ due to the poor domain scores [9, 10, 12, 15, 17, 21–26, 31] (Table 2).

### Stratification of CPG quality

In order to examine which factors may have impacted quality scores in the six domains, we stratified the data on the following variables (guideline area, AGREEII publication date, publication type, working group, comprehensive search or not, fund support or not, and evidence-based or not) in Table 3. We didn’t find the difference in six domains quality related to publication year of AGREEII (before or after 2010). Meanwhile, guidelines published in guideline databases were significantly have a higher scores than that in journals. The scores from CPGs developed by medical societies were higher when compared with individuals for the following items: Scope &Purpose, Stakeholders, Rigour, and Applicability. If CPGs were evidence-based, those three domains (Rigour, Applicability and Editorial independence) would have a higher scores. Apart from above, we found no differences in the rest of the comparisons.

**Table 3:** Mean (±SD) AGREEII scores by subgroups

Subgroups	Scope & Purpose	Stakeholders	Rigour	Clarity	Applicability	Editorial Independence
Year of publication						
≤ 2010 (*n* = 12)	57.17 ± 15.41	26.67 ± 14.93	35.92 ± 19.77	61.00 ± 11.4	32.08 ± 16.62	25.67 ± 25.98
> 2010 (*n* = 10)	45.30 ± 20.18	20.20 ± 10.53	27.80 ± 18.80	51.60 ± 13.34	25.67 ± 25.98	34.30 ± 26.39
*P* values	0.146	0.249	0.337	0.096	0.247	0.451
Publication Type						
Journal (*n* = 16)	44.72 ± 15.14	18.33 ± 7.88	25.72 ± 17.65	53.94 ± 12.35	24.17 ± 8.30	24.83 ± 22.83
Database (*n* = 6)	68.17 + 14.74	38.5 + 12.93	47.67 + 11.84	62.33 + 14.43	45.5 + 12.49	45.17 + 26.63
*P* values	0.003	0.01	0.004	0.239	0.007	0.134
Type of development group						
Individual (*n* = 4)	50.00 ± 16.79	22.25 ± 6.95	30.75 ± 24.42	50.00 ± 13.29	27.75 ± 11.47	26.00 ± 18.17
Medical society (*n* = 18)	52.17 ± 19.07	24.06 ± 14.39	32.59 ± 18.85	58.22 ± 12.74	29.44 ± 14.17	30.39 ± 27.70
*P* values	0.829	0.718	0.896	0.318	0.808	0.707
Systematic search						
No (*n* = 14)	47.57 ± 16.55	19.79 ± 10.70	29.50 ± 17.86	55.43 ± 11.51	26.79 ± 113.64	22.00 ± 27.5
Yes (*n* = 8)	59.13 + 20.01	30.63 + 15.07	37.00 + 22.5	59.00 + 15.70	33.25 + 13.02	42.88 + 17.03
*P* values	0.190	0.1	0.428	0.585	0.288	0.04
Financial						
No (*n* = 14)	46.08 ± 15.97	19.23 ± 9.74	25.31 ± 18.73	52.31 ± 10.78	23.77 ± 8.63	15.08 ± 17.59
Yes (*n* = 8)	60.00 ± 19.21	30.22 ± 15.39	42.22 ± 16.22	63.11 ± 13.68	36.89 ± 15.85	50.56 ± 21.48
*P* values	0.46	0.97	0.51	0.91	0.10	0.18
Evidence-based						
No (*n* = 13)	46.08 ± 15.97	19.23 ± 9.74	25.31 ± 18.73	52.31 ± 10.78	23.77 ± 8.63	15.08 ± 17.59
Yes (*n* = 9)	60.00 ± 19.20	30.22 ± 15.39	42.22 ± 16.22	63.11 ± 13.68	36.89 ± 15.85	50.56 ± 21.48
*P* values	0.09	0.08	0.04	0.07	0.04	0.00

### Discussion

We conducted a comprehensive assessment of the quality of CPGs for constipation. In general, these guidelines existed many deficits. Most of the guidelines had a low score in the following (domain 2, domain 3, domain 5 and domain 6). Table 4 showed that the scores results when compared with international CPGs level [33].

**Table 4:** A comparison of domain scores between these 22 CPGs and international level (%)

Domain	Scope and purpose	Stakeholder involvement	Rigor of development	Clarity of presentation	Applicability	Editorial independence
Constipation mean scores	52	24	32	57	29	30
International mean scores	64	35	43	60	22	30

According to the results, the mean score of domain 3 received only 32.23 %. Methods of the search and the criteria for choose evidence must be clearly described. Meanwhile, the contents of health benefits and risks, externally reviewed by experts should be provided. In order to improve the score of domain 3, particular attention should be paid in above shortcomings.

There were only 2 CPGs included guideline developing experts in the panel [11, 18]. What’s more, no patients was invited to participate in the development term. The domain 5 “applicability” have an important role in the CPGs promotion, it should provide advice and/or tools on how the recommendations can be put into practice. These low scores reflect that CPG producers remain have much work to be done to improve guideline applicability.

Lastly, the scores in the domain 6 were less than 30 %. Many guidelines are developed with external funding, the name of the funding body and a statement that the funding body did not influence the content of the guideline should be explicit consideration [34]. What’s more, there should be a clearly declaration that competing interests of guideline development group members have been recorded and addressed. Therefore, conflict of interests need to be clearly stated.

There are two guidelines which we want to recommend strongly due to their high overall quality developed by Registered Nurses Association of Ontario-Professional Association (RNAOPA) [11] and an Italian guideline by the National Collaborating Centre for Women‘s and Children‘s Health (NCC-WCH) [18]. The detailed recommendations were listed in Table 5. Eight of twenty-two guidelines can be reported with provisos and alterations [13, 14, 16, 19, 20, 27, 28, 30], while the remaining 12 CPGs could not be recommended because most domain scores below 30 % [9, 10, 12, 15, 17, 21–26, 29–31].

**Table 5:** The detail recommendations information of 2 highly guidelines

Title	Items	Recommendations
Prevention of constipation in the older adult population [11]	Practice Recommendations	Assess client history (dietary fibre, medications), Physical activity, Evaluate client response for ongoing interventions
	Education Recommendation	Comprehensive education programs
	Organization & Policy Recommendations	Establish an interdisciplinary team
Constipation in Children and Young People: Diagnosis and Management of Idiopathic Childhood Constipation in Primary and Secondary Care [18]	History-taking	Stool patterns, Symptoms associated with defecation, History, Timing of onset of constipation and potential precipitating factors, Passage of meconium, Growth and general wellbeing, Symptoms in legs/locomotor development, Abdomen, Diet and fluid intake
	Physical examination	Inspection of perianal area: appearance, position, patency, etc.; Abdominal examination; Spine/lumbosacral region/gluteal examination; Lower limb neuromuscular examination including tone and strength, reflexes
	Laxatives	Macrogols (Polyethylene glycol3350 + electrolytes), Osmotic laxatives (Lactulose); Stimulant laxatives (Sodium picosulfate, Bisacodyl, Senna)
	Diet and lifestyle	Infant formula, weaning, insufficient fluid intake; Adequate fluid intake; Adequate fibre
	Clinical investigations	Endoscopy, Test for coeliac disease and hypothyroidism, Test for coeliac disease and hypothyroidism, plain abdominal radiograph, Transit studies, Rectal biopsy, Ultrasound; biofeedback; Antegrade colonic enema procedure

However, our evaluation has several limitations. First, AGREEII rarely suggest how guidelines should select topics. To be useful, guidelines should address the challenges that clinicians face in practice, but developers may exclude clinically important topics when available evidence does not meet minimum standards. Second, inclusion criteria have a language restriction (English and Chinese), language search bias might happen. Third, we used only the AGREEII instrument evaluated the CPGs other than instruments may bring some selection bias [35]. AGREEII instrument have been introduced from 2010, frankly speaking, guidelines published before 2010 did not have access to AGREEII to comply with it. Unfortunately, there is no difference when we compare the six domains quality before and after 2010. We can find even if methodological requirements for CPGs are reported comply with these remains unsatisfactory. What’s more, how to spread the CPGs preferable is essential for clinical practice [36]. Through above specific methodological quality analysis, which can effectively promote the development of future constipation CPGs.

## Conclusions

The results find that the quality of CPGs for constipation is poor. Guideline quality may be improved if we comply with the AGREEII instrument.
